# Recurrent self-limiting abdominal pain with bowel wall edema misdiagnosed as gastroenteritis: a case report of C1-inhibitor-deficient hereditary angioedema

**DOI:** 10.3389/fmed.2026.1818323

**Published:** 2026-04-27

**Authors:** Binlong Zhang, Haocheng Zhao

**Affiliations:** 1 Department of Gastroenterology, Yueqing People’s Hospital, Yueqing, Zhejiang, China; 2 Department of Hematology, Yueqing People’s Hospital, Yueqing, Zhejiang, China

**Keywords:** abdominal attack, bowel wall edema, C1 esterase inhibitor deficiency, complement C4, hereditary angioedema

## Abstract

Hereditary angioedema (HAE) due to C1 esterase inhibitor (C1-INH) deficiency is a rare bradykinin-mediated disease characterized by recurrent episodes of angioedema. Gastrointestinal manifestations can resemble an acute abdomen and are often misdiagnosed, contributing to delayed recognition. We describe a 35-year-old man with two previous self-limited episodes of abdominal pain with diarrhea over the preceding year, beginning in August 2024, each lasting approximately five days, both of which were diagnosed as acute gastroenteritis and treated with symptomatic intravenous therapy. He presented with acute paroxysmal right lower quadrant abdominal pain and watery diarrhea, without fever, nausea, or vomiting. Contrast-enhanced abdominal computed tomography revealed segmental ileal wall edema with ascites, showing slight progression on repeat imaging later the same day. Laboratory analysis demonstrated leukocytosis with neutrophilia and significantly elevated D-dimer levels. Complement testing showed a significantly reduced C4 concentration (0.08 g/L) and a normal C3 level (0.84 g/L), raising suspicion of HAE. Further evaluation confirmed profoundly decreased functional C1-INH activity (<7%) and a reduced C1-INH antigen level (18.89 μg/mL), establishing a diagnosis of type I HAE. The patient improved following supportive and antispasmodic treatment, and no HAE-specific bradykinin-targeted therapy was administered during this admission. This case emphasizes that recurrent, self-limited abdominal pain accompanied by imaging evidence of transient small-bowel wall edema and ascites should prompt consideration of abdominal HAE. Early assessment of complement C4 is a practical screening strategy that enables timely confirmatory testing and minimizes the likelihood of repeated misdiagnosis.

## 1 Introduction

Hereditary angioedema (HAE) is a rare yet potentially life-threatening disease characterized by recurrent episodes of nonpitting, nonpruritic swelling affecting the skin, gastrointestinal tract, and upper airway (1, 2). The most prevalent form results from C1 esterase inhibitor (C1-INH) deficiency caused by pathogenic variants in the *SERPING1* gene (1, 3). Although HAE is classically inherited in an autosomal dominant manner, approximately 25% of cases are associated with de novo mutations; therefore, family history may be absent in a subset of patients. Type I HAE accounts for the majority of cases and is characterized by reduced C1-INH antigen levels and diminished functional activity, whereas type II HAE is distinguished by normal or elevated antigen levels and impaired function (1, 2). In comparison to histamine-mediated angioedema, HAE attacks arise from excessive bradykinin production and typically do not respond to antihistamines, corticosteroids, or epinephrine (1, 4, 5).

Abdominal manifestations occur in a substantial proportion of individuals with HAE and may present with severe cramping pain, nausea, vomiting, or diarrhea (1, 2). However, HAE remains an extremely rare cause of abdominal complaints in general clinical practice. Accordingly, more common causes of acute abdominal pain, including appendicitis, should still be considered and excluded when clinically indicated. Owing to their nonspecific nature, these gastrointestinal symptoms are frequently misattributed to conditions such as infectious gastroenteritis, inflammatory bowel disease, appendicitis, or other causes of acute abdomen (1). Radiologic findings, including transient bowel wall edema and ascites, can provide useful diagnostic clues; however, these findings may still be misinterpreted as enteritis, which can contribute to delayed recognition of the underlying disorder (1).

Assessment of complement C4 is widely considered a practical initial screening test for suspected HAE, as persistently low levels are commonly observed in C1-INH-deficient disease (6). A definitive diagnosis requires demonstration of reduced C1-INH functional activity together with decreased (type I) or normal/increased (type II) antigen levels (1, 6). Although genetic confirmation is not required for every case of HAE type I, SERPING1 genetic testing may be helpful in selected patients, particularly when laboratory findings are inconclusive, the medical history is unclear, or distinction from other forms of angioedema is needed. Establishing the diagnosis is clinically important because it enables appropriate disease-specific counseling and subsequent consideration of on-demand therapy, individualized prophylaxis, and prevention of potentially fatal laryngeal edema (7–13).

In this study, we describe a case of type I HAE presenting primarily with recurrent, self-limited abdominal pain and diarrhea that was repeatedly misdiagnosed as acute gastroenteritis, in which characteristic imaging features and significantly reduced complement C4 levels prompted confirmatory C1-INH testing.

## 2 Case presentation

A 35-year-old man presented on 3 August 2025 with acute, intermittent right lower quadrant abdominal pain accompanied by watery diarrhea occurring 4–5 times within 24 h. He denied fever, chills, nausea, or vomiting. There was no family history of hereditary angioedema or recurrent unexplained swelling, and he reported no previous episodes of cutaneous edema involving the face, limbs, or genitalia, nor any history of laryngeal edema. He was not using angiotensin-converting enzyme inhibitors or other medications associated with bradykinin-mediated angioedema. Moreover, his medical history included two previous self-limited gastrointestinal episodes over the preceding year, beginning in August 2024. Each episode consisted of abdominal pain with diarrhea, persisted for approximately five days, and resolved after supportive intravenous fluids and antispasmodic therapy. Owing to the nonspecific clinical presentation, both episodes were diagnosed as acute gastroenteritis and treated with antispasmodic and supportive therapy, without recognition of the underlying cause at that time. A timeline outlining the recurrent attacks, key diagnostic evaluations, and clinical course is provided in Table 1.

**Table 1:** Timeline of clinical events.

Date	Clinical event	Key findings	Initial interpretation/action
August 2024	First episode	Abdominal pain with diarrhea; symptoms resolved within approximately 5 days	Diagnosed as acute gastroenteritis; treated with intravenous fluids and antispasmodic therapy
7 July 2025	Second episode (outside hospital admission)	Recurrent abdominal discomfort; transoral enteroscopy revealed segmental small bowel mucosal edema approximately 80 cm distal to the ligament of Treitz	Interpreted as jejunitis/duodenitis; hereditary angioedema not considered
3 August 2025	Third episode (current admission)	Paroxysmal right lower quadrant abdominal pain with watery diarrhea; contrast-enhanced CT showed ileal wall edema and small-volume free intraperitoneal fluid	Initially diagnosed as acute gastroenteritis, empirical antibiotic and symptomatic treatment were administered.
7 August 2025	Diagnostic turning point	Complement testing revealed significantly decreased C4 with normal C3	Hereditary angioedema suspected; C1-INH testing ordered
After 7 August 2025	Final diagnosis	Profoundly reduced functional C1-INH activity and decreased C1-INH antigen level.	Diagnosis of type I hereditary angioedema confirmed

**Figure 1:**
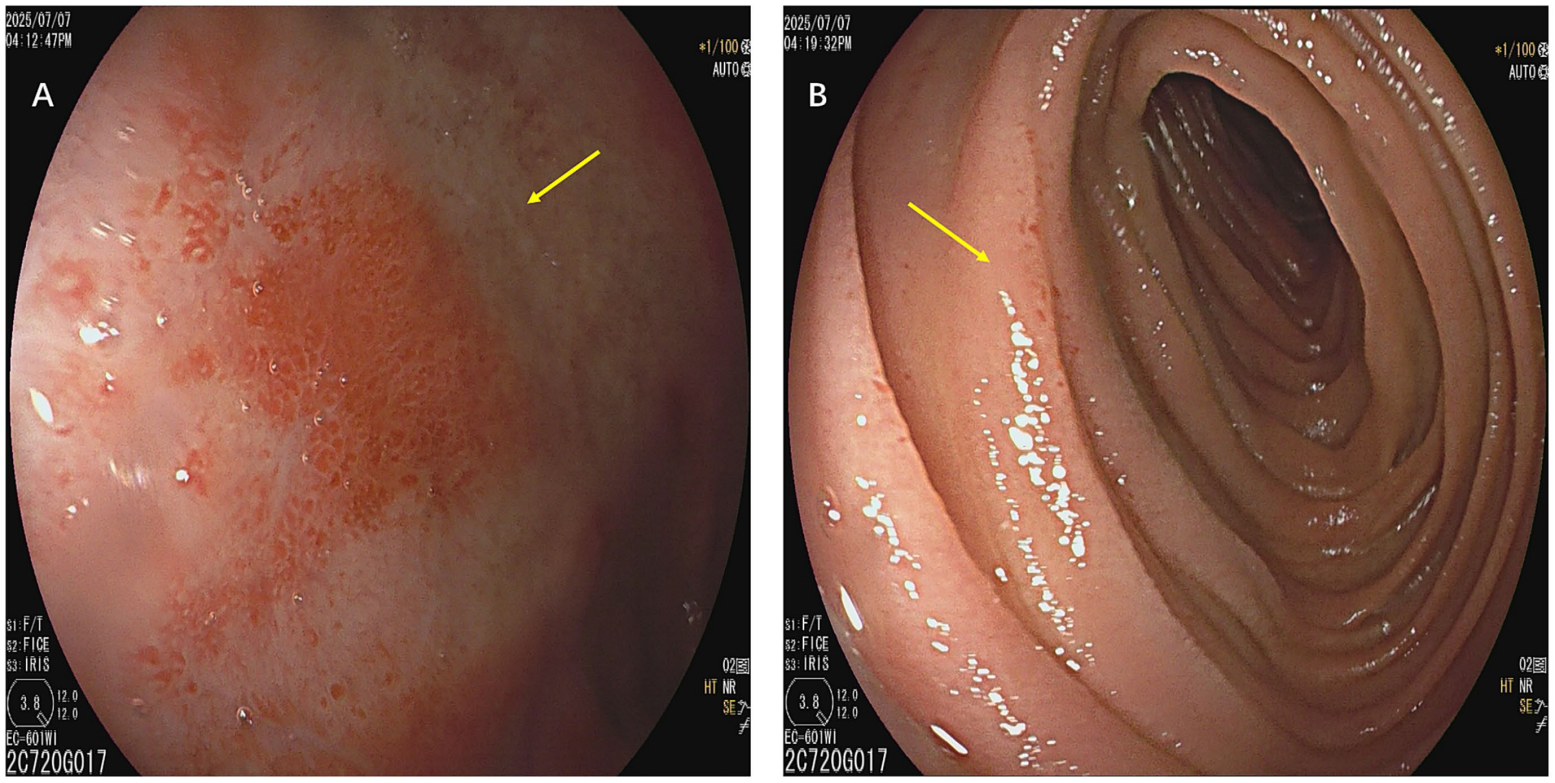
Transoral enteroscopy findings during a recurrent abdominal episode. **(A)** Enteroscopy demonstrates segmental small bowel mucosal edema with pale, swollen mucosa and patchy erythema (arrow). **(B)** A localized edematous mucosal fold with whitish swelling is observed in the jejunum, consistent with transient small bowel edema (arrow).

**Figure 2:**
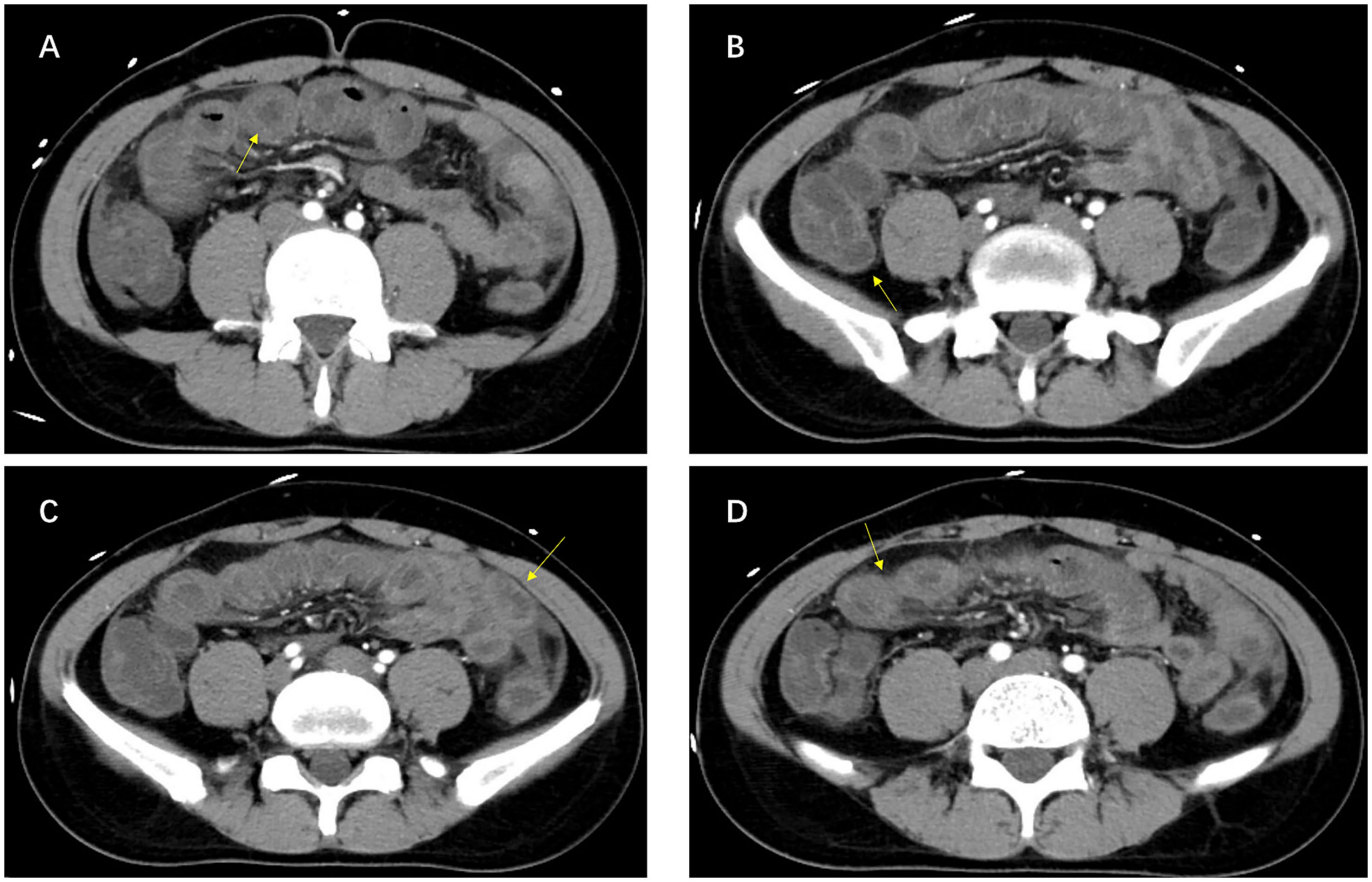
Contrast-enhanced abdominal CT findings during an acute abdominal attack. **(A–D)** Axial contrast-enhanced CT images demonstrate segmental ileal wall thickening with submucosal edema (arrows), accompanied by a small amount of free intraperitoneal fluid.

One month before the current admission, he underwent transoral enteroscopy at another hospital for recurrent abdominal discomfort. The examination revealed segmental mucosal edema of the small intestine approximately 80 cm distal to the ligament of Treitz, which was interpreted as inflammatory enteritis (jejunitis/duodenitis) (Figure 1). No further assessment for hereditary angioedema was performed at that time. In this episode, contrast-enhanced abdominal computed tomography demonstrated ileal wall edema with ascites (Figure 2). Repeat imaging later the same day showed slight progression of bowel wall thickening and a modest increase in ascitic fluid.

Laboratory tests revealed leukocytosis (11.85 × 10^9^/L), neutrophilia (81.9%), mildly elevated C-reactive protein (5.04 mg/L), and significantly elevated D-dimer levels (18.93 μg/mL). Liver and renal function parameters, as well as electrolyte levels, were within normal ranges. Physical examination identified tenderness in the right lower quadrant without guarding or rebound, and no peripheral edema was present.

He was initially treated as a presumed case of acute gastroenteritis and received ceftriaxone together with antispasmodic medication, after which his symptoms improved. However, the pattern of recurrent, self-resolving abdominal attacks combined with imaging evidence of transient bowel wall edema and ascites prompted investigation for noninfectious etiologies. Complement analysis demonstrated a significantly reduced C4 level (0.08 g/L) and a normal C3 concentration (0.84 g/L), strongly suggesting hereditary angioedema with C1 esterase inhibitor deficiency. Confirmatory testing subsequently showed decreased functional C1 esterase inhibitor activity (<7%) along with a reduced antigen level (18.89 μg/mL), establishing the diagnosis of type I hereditary angioedema. Key complement and C1-INH results are summarized in Table 2.

**Table 2:** Summary of key complements and C1-INH findings.

Test	Result	Reference range*	Interpretation
Complement C4	0.08 g/L	0.17–0.48 g/L	Decreased
Complement C3	0.84 g/L	0.80–1.85 g/L	Normal
Functional C1-INH (fC1-INH)	<7.0%	≥58.9%	Significantly decreased
C1-INH antigen level	18.89 μg/mL	81.46–291.29 μg/mL	Decreased

*Reference ranges are based on the testing laboratory standards.

The patient continued to improve with supportive therapy, including antispasmodics (phloroglucinol), probiotics, and gastrointestinal anti-inflammatory agents, and his symptoms resolved. He reported no history of laryngeal edema during earlier attacks. Because the diagnosis was established after his abdominal symptoms had already improved and there was no laryngeal involvement during this admission, no acute HAE-specific therapy was administered in hospital. Before discharge, the patient was informed that HAE attacks, particularly laryngeal edema, can be life-threatening; he was advised to seek immediate emergency medical attention if swelling or respiratory symptoms occurred and was referred for specialist follow-up to arrange access to on-demand therapy and to assess the need for long-term prophylaxis. Because he did not attend the scheduled outpatient follow-up, we have initiated repeated efforts to contact him and to reinforce the need for follow-up evaluation and ongoing counseling regarding the risk of future attacks, including potential laryngeal involvement. At present, long-term outcomes have not yet been systematically evaluated.

## 3 Discussion

This case highlights a diagnostically challenging presentation of abdominal-predominant HAE masquerading as recurrent gastroenteritis-like illness. Although gastrointestinal involvement is common in HAE and may be the predominant manifestation in some patients (1, 2), HAE remains an extremely rare cause of abdominal complaints in routine clinical practice, and more common conditions must be considered first in the evaluation of recurrent abdominal pain. In the present patient, the relatively late onset of symptoms and the apparently isolated abdominal manifestation, without cutaneous or laryngeal swelling, made the presentation atypical for HAE and made early consideration of the diagnosis particularly difficult, especially because HAE more typically presents earlier and often includes additional features such as cutaneous swelling. At the same time, the recurrent self-limiting pattern, together with transient bowel wall edema and ascites, provided a combination of findings that ultimately supported further complement evaluation. Abdominal attacks have been reported in a substantial proportion of patients with HAE and may precede cutaneous manifestations, which can further contribute to diagnostic difficulty (14).

Delayed diagnosis in HAE is a well-documented clinical problem (1, 2). Published cohorts describe prolonged intervals between initial symptom onset and definitive diagnosis, often extending for years (1). Such delays are particularly frequent among patients presenting primarily with gastrointestinal complaints, who may undergo multiple emergency evaluations, imaging procedures, or even unnecessary surgical interventions before the underlying disorder is identified (2). In this case, two previous episodes within one year were treated as presumed infectious enteritis, and endoscopic findings were interpreted as inflammatory changes (Figure 1), illustrating how readily abdominal HAE can mimic common gastrointestinal disorders (1, 3) (Table 1).

Radiologic features offer valuable yet frequently underrecognized diagnostic clues. Transient small-bowel wall edema accompanied by free intraperitoneal fluid, as observed here (Figure 2), is consistent with bradykinin-mediated increases in vascular permeability and submucosal edema (1, 4, 5). In routine clinical settings, however, these findings are often attributed to infectious or inflammatory enteritis, particularly when mild leukocytosis or modest elevations in inflammatory markers are present. During acute abdominal HAE attacks, inflammatory markers may be normal or only mildly elevated; transient leukocytosis and modest C-reactive protein elevation may occur, but marked inflammatory responses should prompt evaluation for alternative causes. Recognition of the episodic and self-limited pattern is therefore essential for distinguishing abdominal HAE from more common causes of acute abdomen (1, 2).

A key diagnostic turning point in this case was complement screening. Persistently decreased complement C4 levels are widely regarded as a practical and accessible screening indicator for C1-INH–deficient HAE (1, 6). Definitive confirmation requires demonstration of reduced C1-INH functional activity together with decreased antigen levels in type I disease (1, 6, 15) (Table 2). Incorporating complement testing into the evaluation of unexplained recurrent abdominal pain, particularly when imaging repeatedly reveals bowel wall edema (Figure 2), may significantly shorten the diagnostic interval (1, 2).

From a clinical standpoint, this case underscores that recurrent, self-limiting acute abdominal episodes should prompt reconsideration of the underlying diagnosis rather than repeated empirical treatment. Even after the diagnosis of HAE is established, abdominal pain should not automatically be attributed to HAE, because alternative and more common causes may still occur and require appropriate evaluation. Disease-specific bradykinin-targeted therapy was not administered during this admission because symptoms improved rapidly with supportive care. Bradykinin-targeted therapies are generally indicated only after the diagnosis of HAE is confirmed, and their use prior to diagnosis would not typically be approved or economically justified. Greater awareness of abdominal HAE among gastroenterologists and emergency physicians is crucial to reduce diagnostic delay, avoid unnecessary antimicrobial use, and ensure timely referral for disease-specific management (12, 13).

## 4 Conclusion

In summary, this case highlights that although common causes account for most presentations of acute abdominal pain, rare conditions such as abdominal HAE should still be considered when the clinical course is recurrent, self-limited, and accompanied by transient bowel wall edema and ascites. In such situations, early assessment of complement C4 may help prompt timely evaluation for C1-INH deficiency. At the same time, clinicians cannot realistically consider every rare disease in every encounter, and future decision-support tools, including artificial intelligence, may help identify uncommon but clinically important diagnostic possibilities in complex cases.

## Data Availability

The original contributions presented in the study are included in the article/supplementary material, further inquiries can be directed to the corresponding author.
